# A Model of Postural Control in Quiet Standing: Robust Compensation of Delay-Induced Instability Using Intermittent Activation of Feedback Control

**DOI:** 10.1371/journal.pone.0006169

**Published:** 2009-07-08

**Authors:** Yoshiyuki Asai, Yuichi Tasaka, Kunihiko Nomura, Taishin Nomura, Maura Casadio, Pietro Morasso

**Affiliations:** 1 The Center for Advanced Medical Engineering and Informatics, Osaka University, Osaka, Japan; 2 Department of Mechanical Science and Bioengineering, Graduate School of Engineering Science, Osaka University, Osaka, Japan; 3 Osaka University of Economics, Osaka, Japan; 4 Italian Institute of Technology, Genoa, Italy; 5 Department of Informatics, Systems, Telecommunications, University of Genova, Genova, Italy; Mount Sinai School of Medicine, United States of America

## Abstract

The main purpose of this study is to compare two different feedback controllers for the stabilization of quiet standing in humans, taking into account that the intrinsic ankle stiffness is insufficient and that there is a large delay inducing instability in the feedback loop: 1) a standard linear, continuous-time PD controller and 2) an intermittent PD controller characterized by a switching function defined in the phase plane, with or without a dead zone around the nominal equilibrium state. The stability analysis of the first controller is carried out by using the standard tools of linear control systems, whereas the analysis of the intermittent controllers is based on the use of Poincaré maps defined in the phase plane. When the PD-control is off, the dynamics of the system is characterized by a saddle-like equilibrium, with a stable and an unstable manifold. The switching function of the intermittent controller is implemented in such a way that PD-control is ‘off’ when the state vector is near the stable manifold of the saddle and is ‘on’ otherwise. A theoretical analysis and a related simulation study show that the intermittent control model is much more robust than the standard model because the size of the region in the parameter space of the feedback control gains (*P* vs. *D*) that characterizes stable behavior is much larger in the latter case than in the former one. Moreover, the intermittent controller can use feedback parameters that are much smaller than the standard model. Typical sway patterns generated by the intermittent controller are the result of an alternation between slow motion along the stable manifold of the saddle, when the PD-control is off, and spiral motion away from the upright equilibrium determined by the activation of the PD-control with low feedback gains. Remarkably, overall dynamic stability can be achieved by combining in a smart way two unstable regimes: a saddle and an unstable spiral. The intermittent controller exploits the stabilizing effect of one part of the saddle, letting the system evolve by alone when it slides on or near the stable manifold; when the state vector enters the strongly unstable part of the saddle it switches on a mild feedback which is not supposed to impose a strict stable regime but rather to mitigate the impending fall. The presence of a dead zone in the intermittent controller does not alter the stability properties but improves the similarity with biological sway patterns. The two types of controllers are also compared in the frequency domain by considering the power spectral density (PSD) of the sway sequences generated by the models with additive noise. Different from the standard continuous model, whose PSD function is similar to an over-damped second order system without a resonance, the intermittent control model is capable to exhibit the two power law scaling regimes that are typical of physiological sway movements in humans.

## Introduction

During human quiet standing, the passive stiffness of the ankle joint, arising from visco-elasticity of the muscle-tendon-ligament system, is lower than the growth-rate of the gravitational toppling torque [1], [2], leaving an upright unstable equilibrium of saddle type which is characterized by a topology of a system's phase space spanned by the position and the velocity providing a convergent motion toward the equilibrium in one direction (a stable manifold) and a divergent motion away from the equilibrium in a different direction like a mountain pass (an unstable manifold). Thus the upright standing posture requires to be stabilized by suitable active control strategies. Many approaches have been investigated for solving this problem and here we focus on the one which has been adopted by the majority of people: a conventional, linear, continuous-time feedback controller based on proportional and derivative feedback (PD control model) [3], [4], [5].

The main challenge is how to compensate the danger of instability induced by the large neural feedback transmission delay, which is of the order of 200 ms [6]. The standard PD model faces a stringent trade-off that leaves narrow margins for the design of the control parameters: the proportional gain must be large enough for supplementing the insufficient ankle stiffness but not too large for avoiding delay-promoted instability. Damping of sway patterns requires rather large values of the derivative gain but again the feedback delay sets a stringent upper bound on this parameter. As we show in the following, the combination of these stability constraints leaves a very narrow area in the *P–D* parameter space where the standard controller is able to provide stability of the upright posture.

We shall contrast the standard controller with a similar feedback PD controller, with the difference that the feedback is switched on and off intermittently, according to a switching mechanism defined in the phase plane. We aim at demonstrating that the intermittent, non-linear controller is more robust than the linear, continuous controller by showing that the stability region in the parameter space is much larger in the former case than in the latter one and, in particular, much lower values of the feedback parameters are required. This control model further expands previous work on the intermittent nature of posture control [7], focusing in particular on a formal stability analysis of such non-linear, delayed feedback control system by means of Poincaré maps.

Moreover, we shall also compare the two models in the frequency domain by looking at the scaling properties of the PSD (Power Spectral Density) of the sway patterns generated by the two models in comparison with biological patterns. It is known indeed [8], [9], [10] that the PSD function of natural sway, if plotted in a log-log scale, can be well fitted by two linearly scaled regimes (or three if very low frequencies are included). That is, the PSD in each regime can be approximated as *f* ^−α^, where *f* is the frequency in Hz and α is the scaling factor. In the lower frequency band (0.01 Hz<*f*<0.2 Hz) the scaling factor is about 1.5, and in the higher band (*f*>0.2 Hz) it is about 3.

## Methods

### Four different controllers of the inverted pendulum model of human standing

In this study, the human upright posture is simply modeled by the motion of an inverted pendulum as
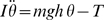
(1)where *I* represents the moment of inertia of human body around the ankle, *θ* the tilt angle, *g* the gravity acceleration, *m* the body mass, *h* the distance from the ankle joint to the body CoM (Center of Mass), *T* the ankle torque, and 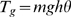 the gravitational toppling torque. The ankle joint torque *T* is modeled as
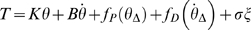
(2)where Δ is the neural transmission delay, 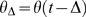 and 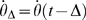. The first two terms on the right hand side of the equation represent passive feedback torques, with no time delay, related to the intrinsic mechanical impedance of the ankle joint (*K* and *B* are the passive stiffness and viscosity parameters, respectively); the third and fourth terms represent the active neural feedback torques that are determined as functions of delay-affected tilt angle and angular velocity, respectively; the last term is a noise torque, modelled as an additive Gaussian white noise ξ(*t*) of intensity *σ*. By combining eq. 1 and eq. 2 we obtain a delay differential equation (DDE):
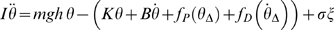
(3)


**Figure 1:**
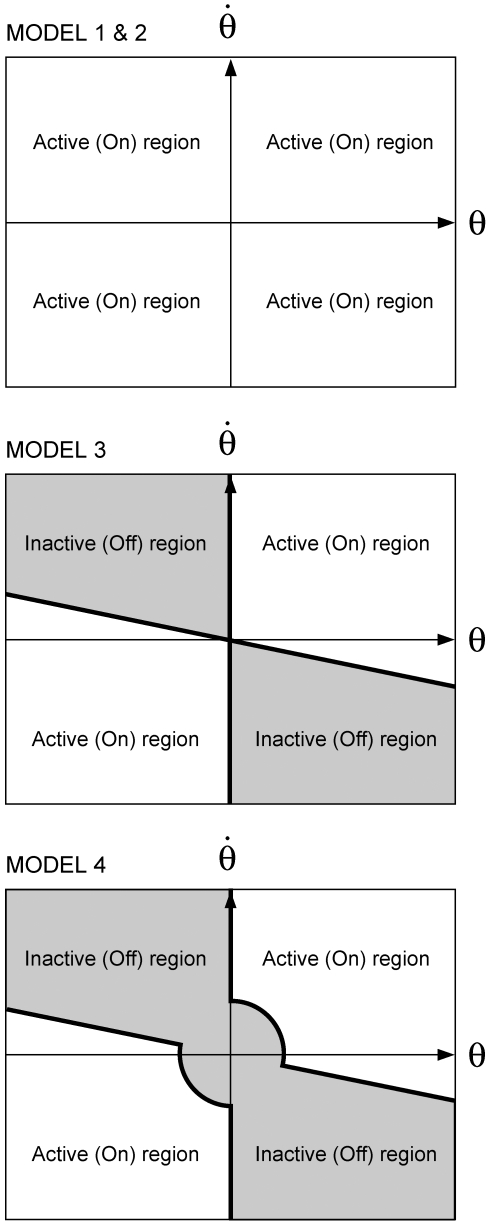
Characterization of the 4 control models in the phase plane (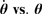). In Models 1 and 2 the control is active in the whole plane. The shaded areas in Models 3 and 4 identify the areas where the control is switched off.

In the following we consider four different implementations of the active controllers *f_P_* and *f_D_* and analyze the corresponding properties and performance. In Models 1 and 2 the active feedback is linear and continuous in time. In Models 3 and 4 the active feedback is non-linear and intermittent. Figure 1 shows, for the four control models, the distribution of active and inactive regions in the phase plane (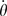 vs. *θ*).

#### Model 1

This model uses a PD linear controller with no time delay (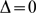):
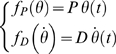
(4)For the system to be asymptotically stable it is necessary and sufficient that, whatever the noise level and the derivative gain *D*>0, *K+P>mgh*. The two eigenvalues are real if *B+D*>2[(*K+P*−*mgh*)*I*]^1/2^ and complex otherwise.

#### Model 2

This model uses a PD controller with time delay Δ:
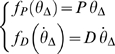
(5)


In this case, the previous condition on the proportional gain is still necessary but is not sufficient. As demonstrated in the Appendix, two additional conditions must be satisfied by the proportional and derivative gains, yielding a set of three conditions to be satisfied by the feedback controller for gaining the asymptotic stability of the upright posture:
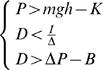
(6)


**Figure 2:**
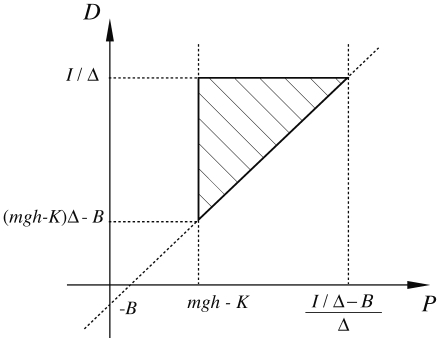
In the plane of proportional and derivative parameters (*P* and *D*, respectively) of the model 1 and model 2 feedback controllers, the figure identifies the region of stability (shaded triangle). Body parameters: *m* (mass); *I* (moment of inertia); *h* (distance of the center of mass from the ankle); *K* (intrinsic stiffness); *B* (intrinsic viscosity); *mgh* (gravity toppling rate). Controller parameters: *P*, *D*, Δ (delay of the feedback loop). As Δ decreases, the triangle increases its area and tends to fill the whole first quadrant to the right of the critical value *mgh-K*. As Δ increases the triangle decreases its area and vanishes when it reaches the value 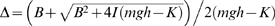.

In the *P–D* parameter plane this identifies a triangle that limits the set of admissible values for the feedback parameters (see Fig. 2). When 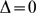, Model 2 is equivalent to Model 1. As Δ decreases, the triangle increases its area and tends to fill the whole first quadrant of the *P–D* plane to the right of the critical value *mgh-K*. On the contrary, as Δ increases the triangle decreases its area and vanishes when it reaches a critical value 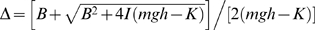 which is a function of the physical parameters of the system (*m,h,I,B,K*). In this study, we consider a physiologically plausible value of 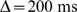 [6], which is fixed throughout the study and is less than the critical value, providing the triangular stable area in the *P–D* plane. A loss of stability of the upright posture occurs when 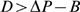 is broken via a Hopf bifurcation, which is a typical critical phenomenon that induces a stable or unstable oscillatory behavior of a dynamical system through instability of an equilibrium state, leading to an unstable oscillation around the upright equilibrium of unstable focus type. Indeed when 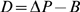, the real parts of the eigenvalues of the linearized equation (eq. 16 in Appendix) vanishes and the upright equilibrium loses its stability.

#### Model 3

In this model the PD controller with time delay Δ is intermittently switched on and off according to a state-dependent mechanism, which divides the phase plane of the pendulum into four regions separated by a negatively tilted line through the origin and the ordinate axis: [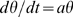] with 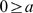 and [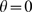]:
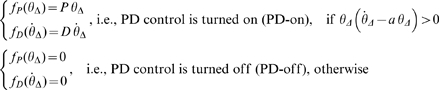
(7)


**Figure 3:**
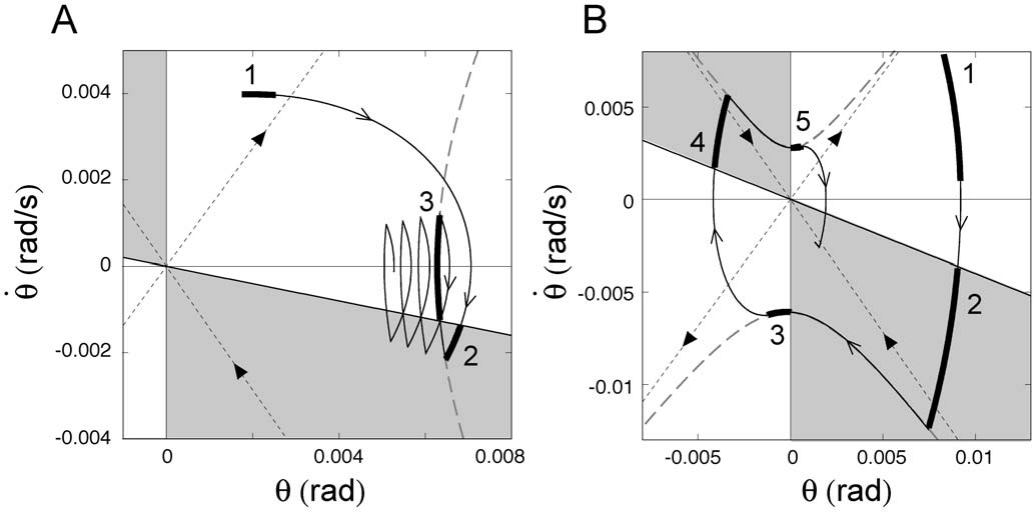
Typical solutions of Model 3 in the phase plane. In each plane, the initial state at *t* = 0 is represented by the thick curve segment labeled “1”. This state segment moves in the phase plane according to the DDE of eq. 3 in number order as labeled. A state of the system at time *t* is represented by the corresponding curve segment whose leading edge is 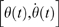 and the tail-end is 
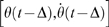
. The PD controller is turned on and off, respectively, if the tail-end of the segment is located in the on (white) and off (gray-shaded) regions in the phase plane. Dotted lines are the stable manifold (arrow heads directing the equilibrium) and the unstable manifold (arrow heads departing away from the equilibrium). A: A typical orbit of eq. 3 when the proportional gain *P* of the PD controller is small. B: A typical orbit of eq. 3 when the gain *P* is large.

Note that the phase space of the DDE of eq. 3 for a nonzero Δ>0 is infinite dimensional, and rigorously speaking, a state of the system at time *t* is a curve segment 
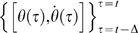
. Therefore the 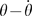 plane cannot be a phase plane of the system. Nevertheless, with keeping carefully this mathematics in mind, we refer to the 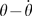 plane as the phase plane. According to eq. 7, the PD-on regions correspond to the first and third quadrants of the phase plane, augmented by two angular slices (in the fourth and second quadrants, respectively) whose amplitude is a function of the switching parameter *a*. The PD-off regions fill the remaining areas of the phase plane. The percentage PD-on vs. PD-off ranges between 50% to 100% as *a* is varied between 0 and −∞. As 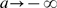, the PD-off region tends to disappear and Model 3 becomes identical to Model 2. Let us illustrate the switching condition for the controller defined by eq. 7 more in detail using Fig. 3: it describes a typical case with the values of *P* and *D* breaking the stability condition 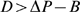 so that the upright equilibrium would be an unstable focus if the PD controller were always turned on. Moreover, the upright equilibrium is also unstable of saddle type if the PD controller were always turned off.

Figure 3A shows a typical solution of Model 3 in the phase plane. The initial state at *t* = 0 is represented by the thick and nearly horizontal curve segment (labelled “1” in Fig. 3A) located at upper left of the first quadrant of the phase plane, representing a slightly forward tilting posture with a velocity falling forward. The right and left edges of the segment are 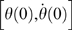 and 
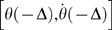
, respectively. This segment moves in the phase plane according to the DDE of eq. 3. A state of the system at time *t* is represented by the corresponding curve segment whose leading edge is 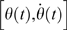 and the tail-end is 
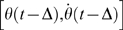
. The condition separated by 
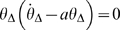
 in eq. 7 implies that the PD controller is turned on and off, respectively, if the tail-end of the segment is located in the on and off regions in the phase plane. Because the tail-end at *t* = 0 is in the on-region in Fig. 3A, the time evolution of the system is governed by 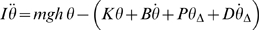 for some time interval, during which the state of the system spirals away from the unstable upright equilibrium of focus type. After a period of time, the leading edge reaches the boundary 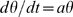 separating the on and off regions (at a point referred to here as R_1_) leaving the tail-end still in the on-region. Then after the time interval Δ, the tail-end also reaches at the boundary 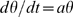 as represented in Fig. 3A by the nearly vertical thick segment (labeled “2”) overflying downward from the boundary 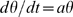 in the off-region, switching the PD controller off. In the off-region, the time evolution of the system is governed by 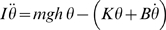 with no PD control for some time interval. Thus the state segment moves upward in the phase plane along a hyperbolic curve (represented by the dashed curve in Fig. 3A) associated with the saddle type upright equilibrium until the tail-end of the segment reaches the boundary 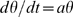 from the off-region side, at which the PD controller is turned on again (the state segment labeled “3”). Then the leading edge of the segment 3 returns to and gets across the boundary 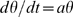 at a point referred to here as R_2_. Similar processes may be repeated as we shall analyze in detail in this study. It is important to note that the leading edge of the state segment labeled “3” in Fig. 3A is located below the orbit connecting the segments 1 and 2. Because of this the point R_2_ is closer to the equilibrium than the point R_1_. If the leading edge of the state segment labeled “3” in Fig. 3A were above this orbit, a subsequent orbit would have returned to the boundary 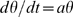 at a more distant point from the equilibrium than the point R_1_.

Figure 3B shows another typical solution of Model 3 when the value of *P* is larger than that used for Fig. 3A. In this case the initial state at *t* = 0 is represented by the thick and nearly vertical curve segment (labelled “1” in Fig. 3B) located at upper right of the first quadrant of the phase plane, representing a forward tilting posture with a velocity falling forward. The leading edge of the segment when the tail-end reaches the boundary 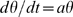 overflies largely into the off-region of the fourth quadrant, due to the large value of *P*, and it goes beyond the stable manifold (the dotted line with arrow heads directing the equilibrium in Fig. 3B) of the saddle equilibrium of the system governed by 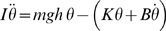. The leading edge when the tail-end reaches the boundary 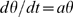 (the curve segment labeled “2” in Fig. 3B) located below the stable manifold moves along a hyperbolic upward-convex curve (the dashed curve in Fig. 3B) directing to the third quadrant of the phase plane to recover the tilting posture. The third quadrant is the on-region, and thus similar but mirror-image processes may be repeated in which the state segment moves from the third to the second quadrant, and then the second to the first quadrant as we shall analyze in this study. Note that the curve segments labelled “3”, “4” and “5” are the states at which the PD controller is turned on, off, and on, respectively.

The stability of this control model, as well as the following one, cannot be computed by means of the standard methods (analysis of the Bode plots, computation of the eigenvalues etc.) due to the non-linearity and intermittency of the controllers. Instead, we shall use Poincaré maps for the orbits in the phase plane that determined by the dynamics of eq. 3 with the control of eq. 7.

#### Model 4

This model is identical to Model 3, with a circular extension of the PD-off region around the origin, i.e. a dead zone in the phase plane:
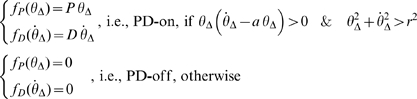
(8)where *r* is the radius of the circular dead-zone. This non-linearity represents the limited sensitivity of the sensors detecting the body tilt and the corresponding falling velocity. Again, the stability of this system will be analyzed by means of Poincaré maps.

The constant parameters used in the simulations are listed in table 1. With these values the passive stiffness *K* is 80% of the critical stiffness *mgh* and thus the upright posture is unstable (saddle) without a suitable active control.

**Table 1:** Model parameters used in the simulations.

*m*	Body mass	60 kg
*I*	Inertia of the body around the ankle	60 kgm^2^
*h*	Distance of the center of mass from the ankle	1 m
*B*	Intrinsic viscosity coefficient	4.0 Nms/rad
*K*	Intrinsic stiffness coefficient	471 Nm/rad (80% of *mgh*)
*g*	Acceleration of gravity	9.81 m/s^2^
Δ	Delay in the feedback loop	0.2 s
*r*	Radius of the dead-zone in the phase plane	0.004 rad-rad/s

### Stability analysis by means of Poincaré maps

The trajectories in the phase plane of the sway movements described by eq. 3, with the control provided by Model 1 or 2, can be a stable or unstable spiral, a stable or unstable node, or a saddle according to the values of the feedback gains *P* and *D* (PD-on flows). Note that the classification of the flows (dynamics) depends on the closed-loop eigenvalues: complex conjugates, with negative real part (flow with stable spiral); complex conjugates, with positive real part (flow with unstable spiral); both negative real (flow with stable node); both positive real (flow with unstable node); both real but with opposite sign (saddle flow). If no control is provided and the intrinsic stiffness is smaller than the critical value, the corresponding PD-off flow is a saddle, which includes a stable and an unstable manifold. If the control is intermittent (Models 3 and 4), the orbits are composed by a combination of PD-on and PD-off flows and the switching function described above automatically selects an orbit along the stable manifold of the latter flow. Therefore, the typical flow in the phase plane determined by Model 3 is a sequence of unstable spiral, followed by a flow along the stable manifold of the saddle and so on, as illustrated in Fig. 3.

The stability analysis of such non-linear dynamics can be carried out by considering a section, transversal to the flow of the system, known as a first return map or Poincaré map. This map can be interpreted as a discrete dynamical system with a state space that is one dimension smaller than the original continuous dynamical system (in our case this implies a reduction from a 2-dimensional problem in the phase plane to a 1-dimensional problem). The stability of the original system can then be reformulated by looking at the fixed point of the map and evaluating its stability.

**Figure 4:**
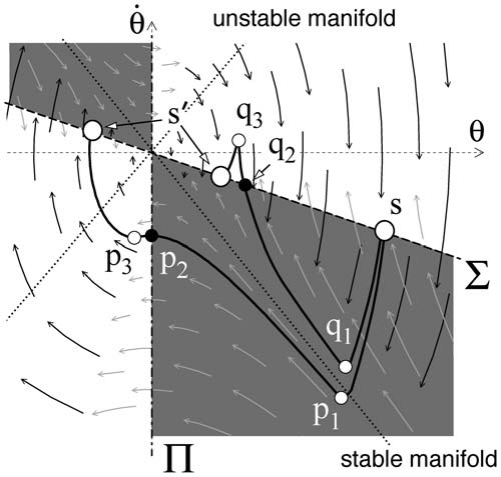
Stability analysis of control model 3 (in the absence of noise) by means of the Poincaré map. Alternation of PD-on and PD-off flows. The lines Σ and Π in the phase plane 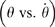 are related to the switching mechanism of the controller. (The shaded areas indicate that the PD-control is switched off.) Σ is also used as the section for the computation of the map. Two typical orbits from Σ to Σ are shown (thick curves) for two different values of the proportional controller gain *P*: *s*→*p*_1_ →*p*_2_ →*p*_3_ →*s'* and *s*→*q*_1_ →*q*_2_ →*q*_3_ →*s'*. The thin lines display the PD-on flows (unstable spiral) and the PD-off flow (saddle with a stable manifold).

With reference to Fig. 4, let us call Π and Σ the two lines in the phase plane that identify the switching function of Model 3 and let us use Σ as the section for evaluating the Poincaré map. Let us denote a state segment at time *t* 
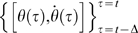
 of the Model 3 as 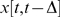: The leading edge and tail-end of the segment are 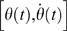 and 
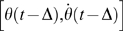
, respectively (see Fig. 3). Let us define 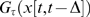 as a flow of the DDE of eq. 3. 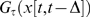 is a function that maps a state segment 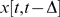 to a time evolved state segment 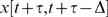 for the time interval *τ* seconds. In Fig. 3, for example, the state segment “1” is mapped to the state segment “2” for a certain time interval *τ*. Characteristics of the flow 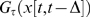 for Model 3 are state-dependent, since the PD controller of the system is switched on and off according to the state-dependent mechanism defined by eq. 7. Let us consider the flow of Model 3 by assuming that the PD controller is always on (as in Model 2), and denote it as 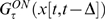. In the same way, we consider the flow of Model 3 by assuming that the PD controller is always off, and denote it as 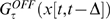. As illustrated in Fig. 3, 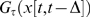 is represented by 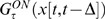 if the tail-end of 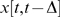 is in the on-region. 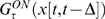 is typically a flow with an unstable focus and referred to as the PD-on flow. If the tail-end of 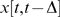 is in the off-region, 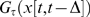 is represented by 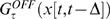 which is a flow with the saddle and referred to as the PD-off flow. Here we approximate the mapping 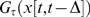 by 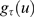 where *u* is the leading edge of the state segment 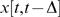 in 2-dimensional phase plane and 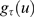 is the leading edge of the state segment 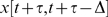 also in the 2-dimensional phase plane, by which characterization of the flow becomes much easier and tractable though less rigorous mathematically. As in 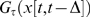, 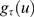 is also represented by PD-on flow and PD-off flow, denoted by 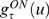 and 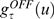, respectively.

The Poincaré map can then be computed by choosing a leading edge *s*∈Σ of a state segment as a starting point of an orbit and tracking it until it reaches Σ again, as a new leading edge *s'* of a time evolved state segment on Σ. As shown in Fig. 4, an orbit from Σ to Σ is always composed of three parts: 1) PD-on part, 2) PD-off part, 3) the second PD-on part. There are two possible patterns according to the specific values of the control parameters. In one pattern (see Fig. 3B), the first part of the orbit (a curve from *s* to 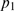 in Fig. 4) is generated by a PD-on flow 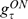, although the leading edge is entering the PD-off region, because the tail-end of the state segment still remains in the PD-on region reflecting the controller takes a time Δ before detecting the switching condition due to the feedback delay. Namely the first part of the orbit is a curve starting from *s*∈Σ (the leading edge of the state segment 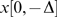 at time *t* = 0), to a point 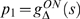 (the leading edge of the state segment 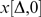 at time 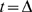). Note that the tail-end of this initial state segment reaches Σ at time 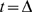 at which the PD control is switched off. Thus the first part of the orbit is identical to the state segment 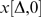. The second part of the orbit brings the leading edge *p*_1_ at time 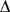 to the leading edge 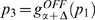 at time 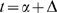, passing through *p*_2_ on Π, with a duration which is composed of two parts: α seconds from 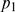 to 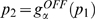 in the PD-off region with the PD-off flow and Δ seconds from 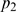 to 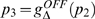 in the PD-on region still with the PD-off flow. As above, the tail-end of the state segment reaches Π at time 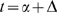, and the PD control is switched on. Once again, note that the orbit from 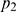 to 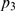 is identical to the state segment 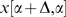 at time 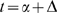. The final part of the orbit brings 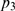 back to the switching line Σ after β seconds: 
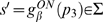
.

The other pattern, shown in Fig. 4, brings *s*∈Σ to *q*_1_, *q*_2_, *q*_3_ and then back to *s'*∈Σ, but with a shorter orbit that does not cross Π (see Fig. 3A). In general, we can define the Poincaré map with the following notation:
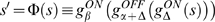
(9)The PD-off flow 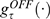 is always the saddle flow with the stable manifold (the dotted straight line with a negative slope on the phase plane in Figs. 3 and 4). The leading edge points *p*_1_ and *q*_1_ when the PD controller is switched off can be close to the stable manifold of the saddle for the choice of the switching function and the value of *P*. In particular, if *p*_1_ or *q*_1_ is exactly on the stable manifold, the state of the system approaches the upright equilibrium directly along the straight line of the stable manifold. Note that if the feedback parameters allow a stable PD-on flow (i.e. *P* and *D* are contained in the triangle stable region of Fig. 2) then also the overall behavior of Model 3 is clearly stable without any need to analyze the Poincaré map. This analysis instead is necessary for evaluating the stability when the PD-on flow is an unstable spiral (focus). For large values of *D* and small values of *P*, the PD-on flow may become an unstable node and in that case the map is not defined, which is the out of range of this study.

For the stability analysis we can restrict the map 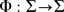 of eq. 9 to the angular values *θ* alone, because the knowledge of points *s* and *s'* on the switching line Σ allows to go back and forth between the sway angle and the angular velocity without any loss of generality:
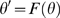
(10)


A map *F* can be obtained numerically, in which a tilt angle 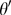 of *s'* on Σ is plotted against a tilt angle *θ* of *s* on Σ as a graph. Once we obtain the map *F*, a sequence of tilt angles at every transverse of the leading edge across Σ can be obtained just by the iterative use of the map. More precisely, for a given initial tilt angle 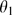 of a leading edge placed on Σ, 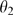 at the subsequent transverse of the leading edge can be obtained as 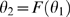. In general, 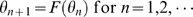. If the upright posture is asymptotically stable, the sequence 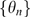 converges to zero as 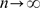. The necessary and sufficient condition for the asymptotic stability of the upright posture (*θ* = 0) is that this posture is a stable fixed point of the map, and this requires that the following condition is satisfied:
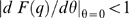
(11)


The orbits generated by Model 4 in the phase plane are the same as those generated by Model 3 as long as the state vector remains outside the dead-zone. However, even in the absence of noise, the control is generally unable to asymptotically drive the system to the upright equilibrium in a stable way. Rather, we should observe a bounded stability, typically with periodic attractors. However, if the size of the dead zone is not too large, in particular if the linear approximation of the sway angles is still valid, then we can expect that the areas of stability in the parameter space for Models 3 and 4 are basically the same.

### Simulation of the inverted pendulum DDE

In the simulations, the DDE of eq. 3 is numerically integrated by using the forward Euler method, with time step Δ*t* = 0.001 s. More precisely, the second order equation of motion is reformulated as the following ordinary delay differential equation:
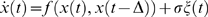
(12)where 
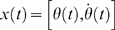
, 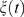 is a normal random process, σ is the corresponding amplitude, and Δ is the feedback delay time. By defining the following discrete normal white noise as a sequence of independent increments of the standard Wiener process (which is an integral of 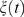) between successive discrete time instants 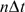 and 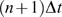 for nonnegative integer *n*:
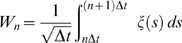
(13)for which 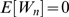 and 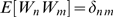, we can rewrite eq. 12 in a discrete form as follows:
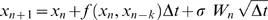
(14)where 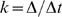. See Appendix for some details. This yields practically a 400-dimensional discretized system for the time delay 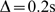. The initial state was set as 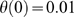, 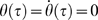 for 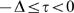. The transient affected by this initial condition was discarded for steady state analyses.

## Results

**Figure 5:**
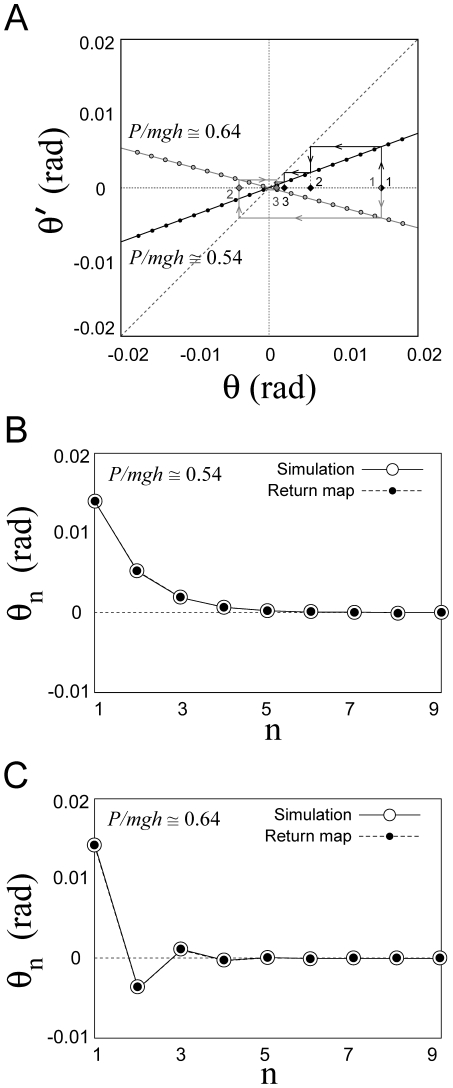
Poincaré map *θ' = F*(*θ*) and its dynamics. A: Two examples of numerically obtained Poincaré map for two different values of *P*. Representation of the return map was restricted to the angular values: *θ* to *θ'*. For each map, an initial tilt angle 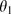 of a leading edge placed on Σ is given, and the subsequent transverse angles of the leading edge across Σ are obtained by 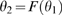 and 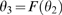. B and C: A sequence of the tilt angles when the state of the system passes through the section Σ obtained by iterative use of the map in the panel A (filled points) and by the DDE simulation (open circles) for Model 3 with *a* = −0.4 s^−1^, and they showed a good agreement. The sequence toward the equilibrium of the sway angle is monotonic in B (*P/mhg* = 0.54) and oscillatory in C (*P/mhg* = 0.64).

The control models introduced in the previous sections were simulated in a systematic way by using different combinations of the control parameters (*P, D, a*). The first issue we wished to address was the robustness of Model 2 (continuous control) vs. Model 3 (intermittent control). To this end, the Poincaré map *θ'* = *F*(*θ*) was obtained for different combinations of the control parameters (*P, D, a*). Figure 5A shows two examples of the Poincaré map for a value of the switching parameter *a* (*a* = −0.4 s^−1^) with a sequence of the tilt angles generated by iterations of each map from an initial tilt angle. The maps could be well approximated by straight lines: the negative slope line describes the convergent dynamics for given values of the parameters *P* and *D* corresponding to the sequence *s*→*p*_1_ →*p*_2_ →*p*_3_ →*s'* in Fig. 4 (see also Fig. 3B); the positive slope line for a smaller value of *P* corresponds to *s*→*q*_1_ →*q*_2_ →*q*_3_ →*s'* in Fig. 4 (see also Fig. 3A). Figures 5B and 5C shows that the iterative use of the map depicted in Fig. 5A generates a convergent sequence of values that have a good agreement with the DDE dynamics, confirming that the Poincaré map can be used practically to analyze the dynamics of Model 3. Note that the convergent sequence of the tilt angles observed repeatedly on the Poincaré section Σ is monotonic if the slope of the map is positive, and it is oscillatory if the slope of the map is negative.

**Figure 6:**
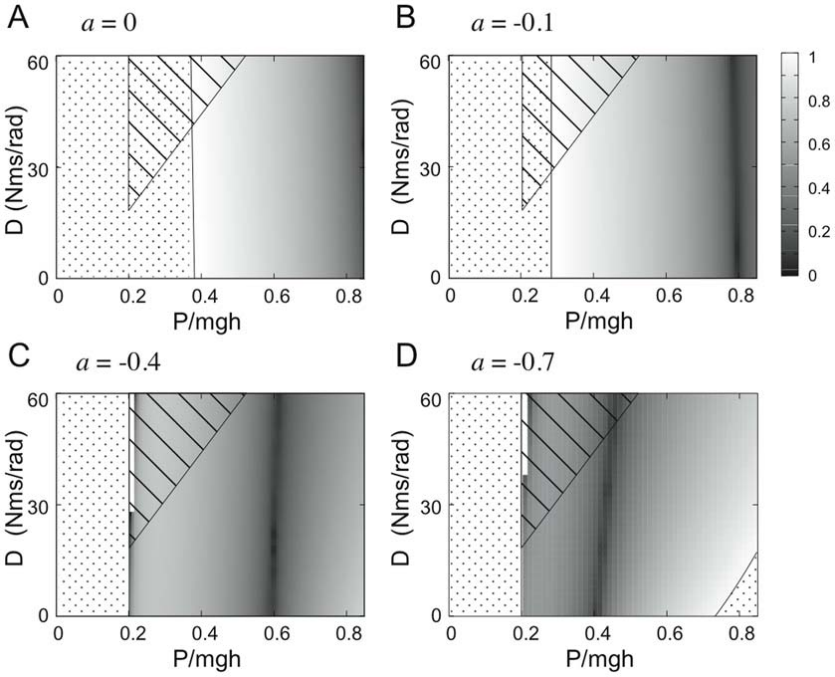
Comparison of the stability region in the *P–D* plane for the control Models 2 and 3. The horizontal axis is normalized with respect to the critical stiffness (*mgh*) considering that the intrinsic stiffness is 80% of that value. The stability region of Model 2 is the striped triangle. The stability region of Model 3 is the grey-shaded area, with a gray intensity which is a function of the absolute slope of the Poincaré map: |*dF*/*d*θ|_θ = 0_ : the darker the shade the quicker the recovery of upright equilibrium. |*dF*/*d*θ|_θ = 0_ = 0 is maximal stability; |*dF*/*d*θ|_θ = 0_ = 1 is neutral stability. Dotted areas correspond to instability (|*dF*/*d*θ|_θ = 0_>1). The four panels show how the stability of Model 3 depends upon parameter *a* which identifies the switching mechanism of Fig. 1. In panels C and D, a small white thin region at the left upper edge of the gray region corresponds to parameter sets in which the equilibrium point is a stable node. Hence in this white region, the Poincaré map cannot be defined though the equilibrium point is stable.

Figure 6 shows the regions of stability in the *P–D* plane for different values of the switching parameter *a*. We find again the stability triangle of Model 2 which clearly does not change with *a*. For Model 3, the figure also shows the distribution in the parameter plane of the absolute slope of the Poincaré map |*dF*(*θ*)/*dθ* |, in which a shading that attributes darker shade represents the more stable conditions.

In general, we can see that the delay-induced instability observed in Model 2 by large values of *P* and small values of *D* is indeed compensated by the intermittent activation of the feedback control. Moreover, for each value of the parameter *a*, there exist optimal sets of *P–D* values that maximize stability. For *P–D* values near the dark linear band of Fig. 6, the points *p*_1_ or *q*_1_ of the orbits when PD control is switched off (see Fig. 4), happen to fall quite close to the stable manifold of the saddle flows, thus leading to the most stable dynamics with “rapid convergence” to equilibrium according to a “sliding motion” along the stable manifold. Moreover, the fact that the dark linear band is almost vertical implies that stability is very little sensitive to the value of *D* and this means that the compensation of the delay-induced instability by means of the intermittent activation of the feedback does not require large values of the derivative gain *D* as occurs with Model 2. In particular, the inverted pendulum can be stabilized even by the zero value of *D* in Model 3.

The optimal value of *P*, given *a* and *D*, is characterized by the fact that the leading edge of the state segment of the PD-on flow, after it enters into the off-region and when the tail-end of the state segment reaches the boundary Σ at which the PD control is switched off, is located exactly on the stable manifold of the saddle flow and this allows the state of the system to approach directly equilibrium without the help of derivative control. If *P* is smaller than the optimal value, then *dF*(*θ*)/*d*θ>0 and the PD-on flow terminates before the leading edge reaches the stable manifold and this yields a monotonic convergent dynamics (Fig. 5B). If *P* is larger than the optimal value, the opposite occurs: *dF* (*θ*)/*d*θ<0 and the PD-on flow terminates after the leading edge reaches the stable manifold and this yields a damped oscillatory convergence to the equilibrium (Fig. 5C).

In any case, Fig. 6 clearly shows that the region in the feedback parameter space where stability can be achieved is much larger for the discontinuous control of Model 3 than the continuous control of Model 2, suggesting that discontinuous control is a more robust control mechanism than continuous feedback control.

**Figure 7:**
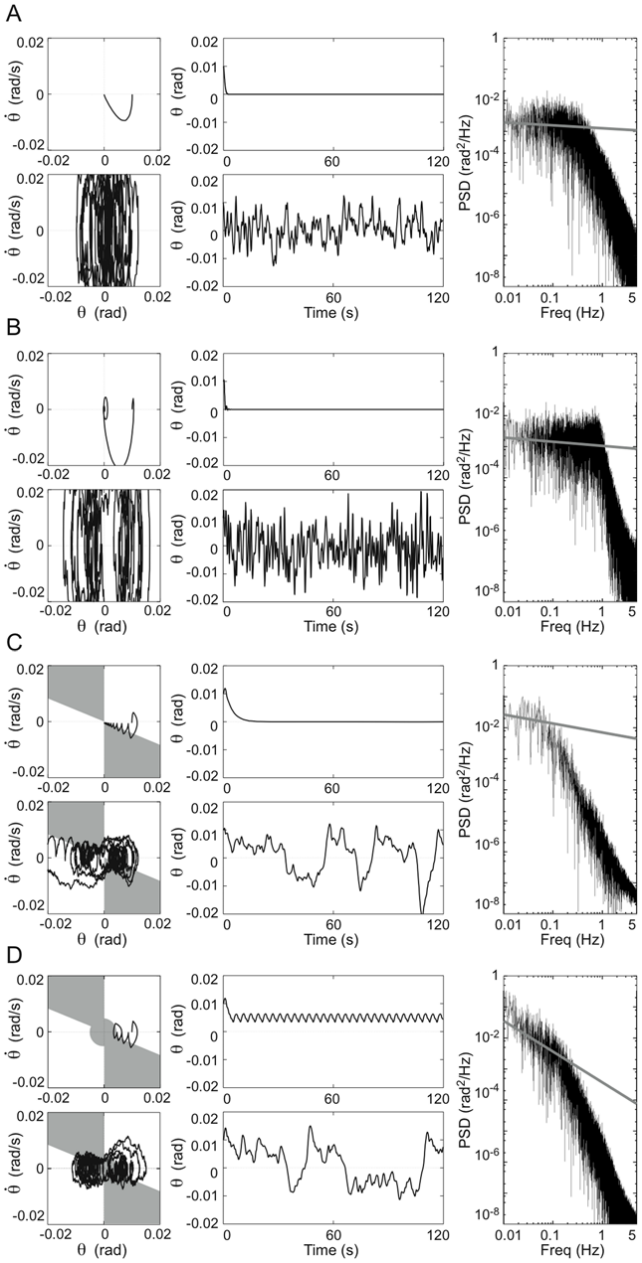
Simulation of the four control models with and without noise: Model 1 (panel A); Model 2 (panel B); Model 3 (panel C); Model 4 (panel D). Each panel shows: 1) trajectories in the phase plane (left-upper part without noise, left-lower part with noise); 2) corresponding angular sway sequences (middle-upper part without noise, middle-lower part with noise); 3) power spectral density for the model with noise (right part). In the shaded areas of the phase planes, PD control is switched off. For Models 1 and 2, the following parameters were used: *P*/*mgh* = 0.8, *D* = 270 Nms/rad, *σ*  = 2 Nm. For Models 3 and 4 the parameters in the PD-on regions were as follows: *P*/*mgh* = 0.25, *D* = 10 Nms/rad, *σ*  = 0.2 Nm, and *a* = −0.4 s^−1^.

**Figure 8:**
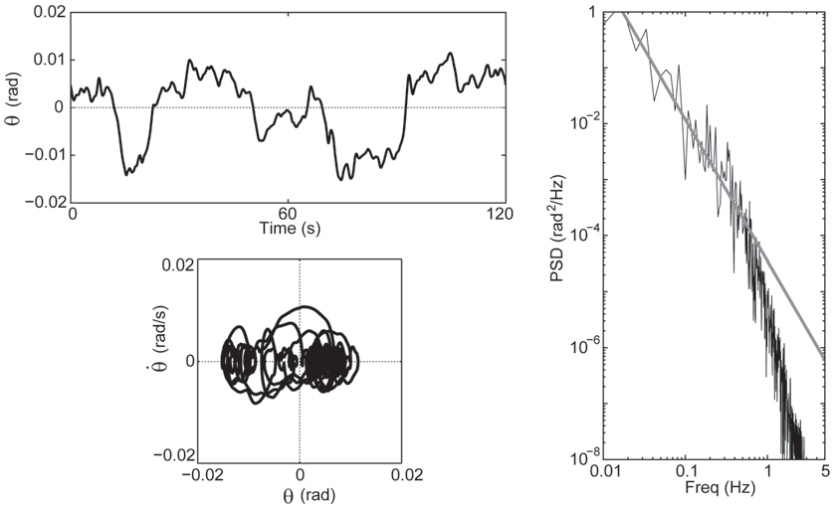
Sample of human postural sway, collected from a subject in quiet standing for 120 s. Left upper panel: Angular sway sequence; Left lower panel: trajectory in the phase plane; Right panel: power spectral density of the angular sway.

Figure 7 shows typical simulated dynamics with and without noise for each of the four models, to be compared with experimental data coming from a typical human subject (Fig. 8. See [11] for the corresponding experimental setup.). Models 1 and 2 are asymptotically stable for large PD gains that are close to the values used in previous studies [3], [4], exhibiting a rapid decay to the equilibrium in the noise free case from the given initial condition and a stochastic sway distribution centered around the upright posture in the presence of noise. Model 3 also shows asymptotic stability with a point attractor at the origin but it requires much smaller values of the *P* and *D* parameters (*P*/*mgh* = 0.8, *D* = 270 Nms/rad for Model 2 and *P*/*mgh* = 0.25, *D* = 10 Nms/rad for Model 3).

Model 4 has two periodic attractors, with a positive and a negative average angular values. In the absence of noise it settles in one oscillatory mode or the other as a function of the initial state of the simulation. The noise induces alternations between these two attractors, which are more prominent than the alternations observed in Model 3 and this agrees with the bimodal angular histograms observed by Bottaro et al [7].

Figure 7 also shows typical power spectra of the four control models, to be compared with the power spectrum of human sway (Fig. 8). In Models 1 and 2, due to the large PD gains, the PSD profile is roughly a second order type without a resonance whereas in Models 3 and 4 we clearly find the two power law scaling regimes typical of human sway. Moreover, Models 1 and 2 require much larger noise intensities to reproduce the physiologically plausible sway amplitude than Models 3 and 4: σ  = 2.0 Nm in Fig. 7A–B and σ  = 0.2 Nm for Fig. 7C–D.

Noise-free DDE simulations of Model 4 for various sets of *P-D-a* parameters show that the stable regions in the *P-D-a* parameter space of Model 4 are the same as those of Model 3, with the difference that the attractor of Model 3 is an asymptotically stable equilibrium point and that of Model 4 is a limit cycle. This fact provides a common basis for understanding the noisy dynamics of both Models 3 and 4. As shown in Fig. 7, the power spectra of Models 3 and 4, if affected by noise, exhibit the two power law scaling regimes that are typical of physiological sway movements. In particular, the first power law scaling factor at the low frequency regime of Model 4 changes depending on the values of *P*, *a*, and the noise intensity *σ* as these parameters determine stochastic occurrences of the slow motions along the saddle manifold. In the noise-free case, two limit cycle attractors coexist in Model 4 and the state point oscillates around one limit cycle or the other.

These oscillatory patterns are characterized by the fact that PD control is switched on for one part of the limit cycle and switched off for the remaining part. The distance between the stable manifold of the saddle dynamics that describes the system's behavior when PD-control is off and the leading edge of the state segment in the phase plane when the PD control is turned off depends on the values of *P* and *a* as we have demonstrated for Model 3. If the distance is small, a small noise added to Model 4 can push the state point moving closely along the stable manifold of the PD-off flow, which is a part of “the noisy limit cycle,” to the opposite side of the phase plane, leading to the stochastic switching from one limit cycle attractor to the other. If the distance is large, a noise of larger intensity is required to induce the alternation between the attractors: the alternation frequency between the attractors tends to increase with noise intensity. However, the alternation occurs most frequently if the distance and the noise intensity match. This could be considered as a type of a stochastic resonance.

**Figure 9:**
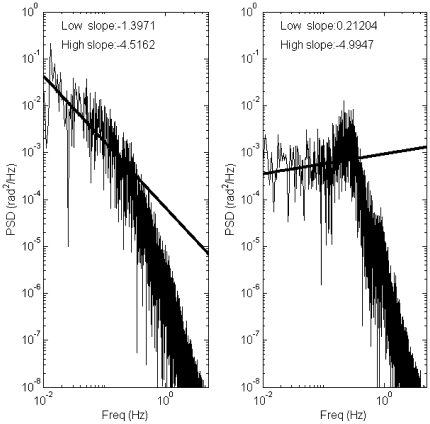
Power spectral density functions (PSDs) of sway data for Model 4 with two different parameter values. Left panel: *P* = 176 Nm/rad (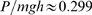), *D* = 10 Nms/rad, *a* = −0.4 s^−1^. Right panel: *P* = 470 Nm/rad (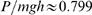), *D* = 10 Nms/rad, *a* = −0.4 s^−1^. For Models 3 and 4 with *a* = −0.4 s^−1^, the optimal the optimal value of *P* for the stability is about 60% of *mgh* (i.e. 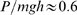) regardless the value of *D*. The values of *P* in the left and right panels are smaller and larger than the optimal value of *P*, respectively.

Figure 9 shows two examples of the PSD for Model 4 with two different values of *P* (*P*/*mgh*≈0.29 for the left panel and *P*/*mgh≈*0.79 for the right panel) and common values of *D*, *a*, and σ (*D* = 10 Nms/rad, *a* = −0.4 s^−1^, σ  = 0.2 Nm). For this value of the switching parameter *a*, the optimal value of *P* for the stability of Model 3 was about 60% of *mgh* regardless the value of *D*, i.e., the dark band was located at 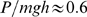 in Fig. 6C. Thus the values of *P* used for Fig. 9 left and right are, respectively, smaller and larger than the optimal value of *P*. As in these two examples, the PSD showed the two power law scaling for smaller values of *P*, and it was more like a second order system and similar to the PSD of Model 2 with or without a resonance for large values of *P*.

**Figure 10:**
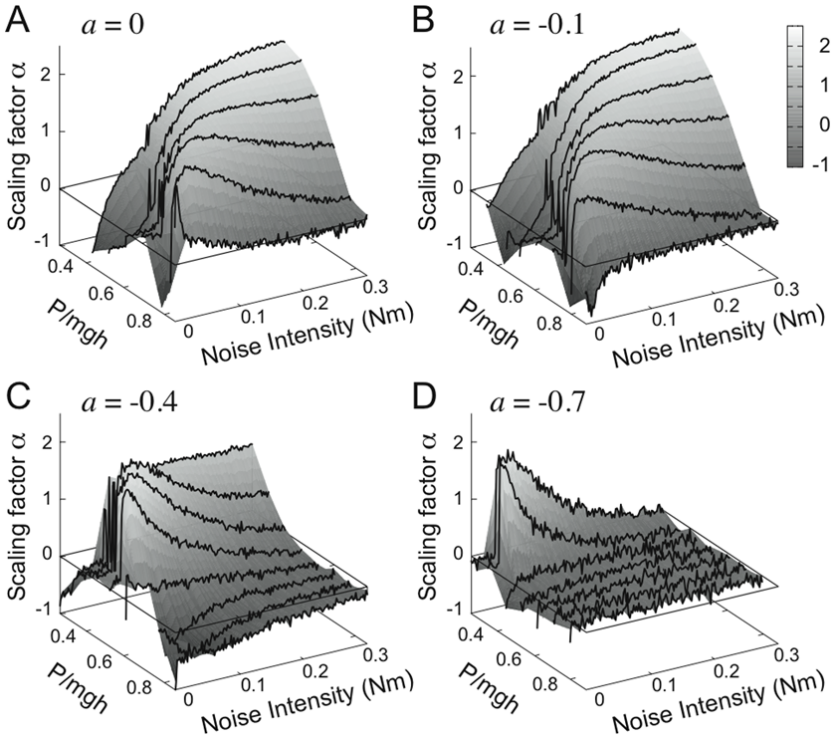
For the feedback control Model 4 (intermittent with dead zone) the figure shows the dependence of the scaling factor *α* of the PSD function upon the following parameters: 1) noise intensity; 2) proportional feedback gain *P* (normalized with respect to the critical stiffness *mgh*); 3) the slope *a* of the switching function. The derivative feedback gain *D* is fixed at the value of 10 Nms/rad. The values of *α*, with appropriate choice of *σ*, *P* and *a*, are comparable with the physiological value which is about 1.5. A: *a* = 0 s^−1^. B:*a* = −0.1 s^−1^. C:*a* = −0.4 s^−1^. D: *a* = −0.7 s^−1^.

Figure 10 shows, for Model 4, the dependence of the scaling factor *α* of the power spectrum of the noisy sway at the low frequency regime, as a function of *σ* and *P* for several *a* values with a fixed low derivative gain *D* at 10 Nms/rad. For a given value of *P, α* tends to be a unimodal function of *σ* when *P* is close to the optimal value taken from the dark band of Fig. 6 for which the corresponding Model 3 exhibits the most stable dynamics (see the unimodal curve of Fig. 10C at 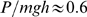 as the function of *σ* as a typical example). The peak value of the unimodal curve is attained when the noise intensity matches the distance between the stable manifold and the leading edge just after the PD control is switched off as described above. If *P* is smaller than the optimal value chosen from the left-hand side of the dark band of Fig. 6, the unimodal curve gradually becomes monotonic increasing function with a saturated value. The peak and the saturated values of *α* are close to or larger than 1.5 depending on the value of the switching parameter *a*: this is close to the physiological scaling factor [8], [9], [10] and the PSD is more or less similar to Fig. 9-left, exhibiting the two power law scaling. On the other hand, the unimodal curve disappears and the curves of the scaling factor *α* as the function of *σ* become almost flat close to zero or even negative if *P* is larger than the optimal value, i.e., if it is taken from the right-hand side of the dark band for which Model 3 shows damped oscillations (refs. Fig. 5C). That is, the PSD is similar to a second order system with or without a resonance (e.g., Fig. 9-right). In particular, the PSD does not exhibit a resonance peak for the value of *P* larger than but close to the dark band, and it shows a small resonant peak if the value of *P* is more away from the dark band and if the noise intensity is small.

## Discussion

The intermittent control strategy explored in this paper is based on the idea that, in order to control the behavior of a system characterized by a saddle-type unstable equilibrium, it is smart to take advantage of the stable manifold of the saddle and focus the active control intervention on the task of keeping the state of the system as close as possible to such manifold by means of a sequence of small, well timed control signals. This is an idea which has been used in different fields, in order to control physical [12], physiological [13], and clinical [14] systems. In Gibsonian terms [15] we may say that, in spite of the instability of the saddle-type equilibrium, the stable manifold of the saddle is an “affordance” that a smart agent is supposed to exploit in order to simplify the control problem and provide a more economical solution: indeed part of the job can be performed by the “environment” not the controlling agent.

An intermittent control mechanism for continuous-time control systems with feedback delay is similar to the “act-and-wait” concept proposed by Insperger [16]. The difference with respect to our model is that the switching mechanism is periodic instead of being event-driven: although such mechanism can be efficient, it leaves open the choice of the switching period and, in any case, there is no hint of biological plausibility of this type of solution in the case of posture control.

One of the results of this study is that the intermittent PD controller can achieve stable equilibrium with very small or even null values of the derivative gain *D*. However, this does not imply at all that the velocity information of the postural sway is not important [5], [17]. On the contrary, the estimate of sway velocity is one of the key sensory information for switching on and off the active control in the intermittent control at the right time. In particular, the distance between the stable manifold of the saddle for the PD-off flow and the point when the PD control is switched off determine the postural stability, and it is determined by the timing of the switching off of the PD control, for which the velocity information is critical. In this regard, the intermittent control model proposed by Bottaro *et al* [7] incorporates an internal model of the body dynamics for generating the active control torques, by which the appropriate amount of the intermittent and brief control torque calculated based on the internal model can locate the state point close to the stable manifold, leading to a more robust stability of the quiet standing.

Another study [18], [19], [20] has investigated the properties of the following DDE:
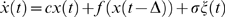
(15)where *x* represents the postural tilt angle, and 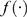 is the 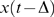 dependent on-off switching function. They have shown that the model has two coexisting limit cycles: noise can induce transitions between the two attractors, resulting in multiple scaling of two point correlation functions. In this regard, the model above reproduces similar dynamics to Models 3 and 4 of this study. An important difference is that eq. 15 is derived by neglecting the inertia term from the equation of motion in order to reduce the analysis to a first order DDE. They justified this by assuming that the system is overdamped, i.e. the ankle viscosity is high enough. However, we showed in Models 3 and 4 that the total viscous torque (including the passive viscosity *B* as well as the “active viscosity” *D*) could be very small and comparable with or even smaller than the inertia torque. Indeed, we have examined experimental sway data to compare the inertia and viscous terms during human quiet stance, confirming that the inertia term should not be neglected. Several features of the postural sway movements suggest indeed the overdamped dynamics: the non-resonant PSD, the non-oscillatory impulse responses to small perturbations, etc. However, our study demonstrates that the same outcome can be obtained by a properly tuned “saddle mechanism”, even with a very small level of ankle viscosity.

We showed that the postural sway with the intermittent activation of the PD controllers can reproduce the multiple power law scaling property of the PSD during human quiet stance when the upright posture is perturbed by white noise with appropriate intensity. Note that there is one-to-one correspondence between each power law regime of the PSD and that of the two point correlation function, since PSD and the two point correlation function are simply interrelated by the Fourier transform [21]. The basic mechanism underlying the two power law scaling regimes in Models 3 and 4 is stochastic switching of the state between left and right halves of the phase plane. In particular, the switching occurs between two coexisting limit cycles. This is similar to what has been shown by Milton and colleagues [18]. Nevertheless, it is worthwhile to note that continuous PD and/or PID models, which are most popular models of the upright postural control, require colored noise whose spectral property is responsible for the power law scaling at the low frequency regime. The discontinuous, intermittent control hypothesis can provide alternative mechanisms to generate physiological postural fluctuation that can be characterized by the power law scaling at the low frequency regime. Moreover, we showed that several physiological parameters, such as the feedback gains of the active controller, the condition determining the intermittent activation of the PD controller, and the noise intensity can change the scaling factor. This suggests that one might be able to perform a model based estimation of these parameter values based on the scaling factor.

This study shows that the intermittent feedback control of the postural dynamics with “the tuned” on-off regions can robustly stabilize the upright posture even with small PD gains. Small gains provide compliant postural dynamics and thus the physiologically plausible small amount of noise can naturally generate the fluctuation during quiet standing [22]. The study may also provide new insight in well studied experimental paradigms during human quiet stance, such as the noise-enhanced sensation and disease-induced abnormal sensation, by examining them with the intermittent control hypothesis. Experimental paradigms with a modification of the sensory dead-zone [23], with noise-intensity dependent changes in the balance control [24], and with rigidity-dependent varied ankle stiffness in patients with Parkinson's disease could be examined with our model. The models analyzed in this study might be able to predict how the corresponding parameters can affect the sway patterns.

In many different paradigms of neural control of movement has emerged the concept that the control patterns might be organized in well-timed, intermittent bursts or chunks: saccadic/tracking eye movements [25], postural sway movements [17], [22], visuo-manual tracking [26], [27], stick-balancing on a finger tip [28], [29], cursive handwriting [30]. Overall, the origin of the such intermittency remains obscure and has, up to now, been viewed mainly as a consequence of neurophysiological internal constraints that limit the computing power of the neural controller. However, there is the alternative possibility that intermittency has a functional role in the control strategy of human subjects: that of maintaining the stability of feedback control despite uncertainties about dynamic properties of the body or manipulated objects and the large neural delays in the transmission of the feedback signals. An example of discontinuous, impulsive control playing a functional role, other than in the postural control analyzed in this study, is in the dynamic stabilization of gait patterns: Yamasaki et al [31] showed that impulsive, well-timed phase resetting in response to external perturbations during the rhythmic motor control of human gait can increase dynamics stability of motions in a better way than conventional, continuous feedback control, affected by large feedback delays.

There are various sources of non-linearity in the neural control of movement (muscle elasticity, hysteresis, joint friction and viscosity, Coriolis coupling) and a high degree of redundancy. When a subject has to perform movements with external mechanical constraints or to manipulate an object, some of the dynamic characteristics of the resulting controlled system are not precisely known by the central nervous system. One of the key issues in studying motor control is to understand how the brain can generate the appropriate command. This question is closely related to a classical problem in robotics where manipulator controllers have to be built in order to achieve similar tasks in such a way that control stability is guaranteed. Such control problems are greatly simplified by the introduction of intermediate “sliding variables” [32], [33]. A sliding variable is a specific combination of the instantaneous error and its time derivative (a particular case of composite variable). By choosing this composite variable so that the implicit differential equation is stable, high order control problems can be reduced to first-order problems, amenable to qualitative feedback strategies, typically organized in intermittent manner.

In the case of postural control 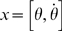 can be considered as a sliding variable, a useful simplification that allows the brain to greatly reduce the dimensionality of the control problem. In fact, the formalization of the control problem by means of eq. 3 is clearly a simplification because the body is not an inverted pendulum and the sliding variables themselves, *θ* and 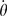, are abstractions that are not directly measurable if we consider the multijoint structure of the body and the “paradoxical” contraction of the gastrocnemii [34]. In this framework, the proposed intermittent control model is an example of how seeking the right kind of simplification is a strategy adopted by the human brain for managing complexity as well by the scientists for analysing the complexity of real problems: We compared two simplified explanations of the same problem and suggested that one explanation is better than the other because is more robust and better explains important features of biological behavior.

### Appendix

#### Linear stability analysis of Model 2

The DDE of the system with the model-2 controller is the following:
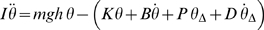
Following Stépán and Kollár [35] we approximate 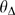 and 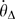 by their first-order Taylor's series expansion
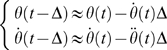
thus yielding
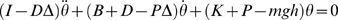
(16)In other words, the delay tends to decrease the apparent inertia and damping of the inverted pendulum but both must remain positive for stability because the eigenvalues solve the following equation:
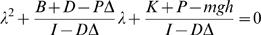
From this we can derive the conditions on the gains of the feedback controller, listed in eq. 6.

#### Euler approximation of a stochastic differential equation

Equation 12 can be interpreted symbolically as an Ito stochastic differential equation
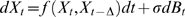
(17)where 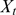 represents the random variable version of 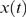, and 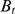 represents the Brownian motion or the standard Wiener process defined for 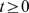. The Euler approximation of eq. 17 with a fixed time step 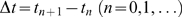 is:
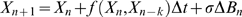
(18)where 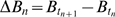 is the random increments of the Wiener process for the time interval between *t_n_* and *t_n_*_+1_, and 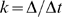. The increments are independent Gaussian random variables with mean 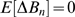 and variance 
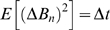
. See [36] for more details. Using a discrete white Gaussian noise of intensity unity, i.e., independent Gaussin random variables 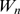 with mean 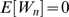, variance 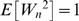, and 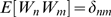 in stead of 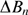, eq. 18 can be rewritten as:
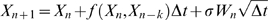
(19)This is equivalent with eq. 14 in the main text. Note that eq. 19 can also be rewritten as:
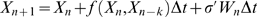
for 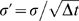. For 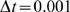 as we utilize in this study, 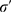 is 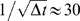 times larger than *σ*. If we conventionally consider 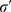 as the intensity of noise (as is the case in some previous studies), the typical values of the noise intensity 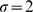 Nm and 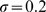 Nm used in this article can be read as 60 and 6, respectively.

## References

[1] Loram I, Lakie M (2002). Direct measurement of human ankle stiffness during quiet standing: The intrinsic mechanical stiffness is insufficient for stability.. Journal of Physiology.

[2] Casadio M, Morasso P, Sanguineti V (2005). Direct measurement of ankle stiffness during quiet standing: Implications for control modelling and clinical application.. Gait & Posture.

[3] Peterka R (2000). Postural control model interpretation of stabilogram diffusion analysis.. Biological Cybernetics.

[4] Maurer C, Peterka R (2005). A new interpretation of spontaneous sway measures based on a simple model of human postural control.. Journal of Neurophysiology.

[5] Masani K, Popovic MR, Nakazawa K, Kouzaki M, Nozaki D (2003). Importance of body sway velocity information in controlling ankle extensor activities during quiet stance.. Journal of Neurophysiology.

[6] Peterka RJ (2002). Sensorimotor Integration in Human Postural Control.. Journal of Neurophysiology.

[7] Bottaro A, Yasutake Y, Nomura T, Casadio M, Morasso P (2008). Bounded stability of the quiet standing posture: An intermittent control model.. Human Movement Science.

[8] Collins J, De Luca C (1994). Random walking during quiet standing.. Physical Review Letters.

[9] Chow C, Collins J (1995). Pinned polymer model of posture control.. Physical Review E.

[10] Chow C, Lauk M, Collins J (1999). The dynamics of quasi-static posture control.. Human Movement Science.

[11] Nomura K, Fukada K, Azuma T, Hamasaki T, Sakoda S, Nomura T (2009). A quantitative characterization of postural sway during human quiet standing using a thin pressure distribution measurement system.. Gait & Posture.

[12] Ott E, Grebogi C, Yorke JA (1990). Controlling Chaos.. Physical Review Letters.

[13] Schiff S, Jerger K, Duong D, Chang T, Spano M, Ditto W (1994). Controlling chaos in the brain.. Nature.

[14] Tanaka G, Tsumoto K, Tsuji S, Aihara K (2008). Bifurcation analysis on a hybrid systems model of intermittent hormonal therapy for prostate cancer.. Physica D.

[15] Gibson JJ (1950). The perception of the visual world.

[16] Insperger T (2006). Act–and–wait concept for continuous–time control systems with feedback delay.. IEEE Transactions on Control Systems Technology.

[17] Loram ID, Maganaris CN, Lakie M (2005). Human postural sway results from frequent, ballistic bias impulses by soleus and gastrocnemius.. Journal of Physiology.

[18] Eurich C, Milton J (1996). Noise-induced transitions in human postural sway.. Physical Review E.

[19] Milton JG, Cabrera JL, Ohira T (2008). Unstable dynamical systems: Delays, noise and control.. Europhysics Letters.

[20] Milton J, Townsend J, King MA, Ohira T (2009). Balancing with positive feedback: the case for discontinuous control.. Philosophical Transactions of Royal Society A.

[21] van der Kooij H, van Asseldonk E, van der Helma FCT (2005). Comparison of different methods to identify and quantify balance control.. Journal of Neuroscience Methods.

[22] Bottaro A, Casadio M, Morasso P, Sanguineti V (2005). Body sway during quiet standing: Is it the residual chattering of an intermittent stabilization process?. Human Movement Science.

[23] Meyer PF, Oddsson LI, De Luca CJ (2004). The role of plantar cutaneous sensation in unperturbed stance.. Experimental Brain Research.

[24] Priplata A, Niemi J, Salen M, Harry J, Lipsitz LA, Collins JJ (2002). Noise-Enhanced Human Balance Control.. Physical Review Letters.

[25] Bahill AT, Stark L (1979). The trajectories of saccadic eye movements.. Scientific American.

[26] Miall RC, Weir DJ, Stein JF (1993). Intermittency in human manual tracking tasks.. Journal of Motor Behavior.

[27] Hanneton S, Berthoz A, Droulez J, Slotine JJE (1997). Does the brain use sliding variables for the control of movements?. Biological Cybernetics.

[28] Cabrera J, Milton J (2002). On-off intermittency in a human balancing task.. Physical Review Letters.

[29] Cabrera J, Milton J (2004). Human stick balancing: Tuning Le'vy flights to improve balance control.. Chaos.

[30] Morasso P, Ivaldi MFA, Ruggiero C (1983). How a discontinuous mechanism can produce continuous patterns in trajectory formation and handwriting.. Acta Psychologica.

[31] Yamasaki T, Nomura T, Sato S (2002). Possible functional roles of phase resetting during walking.. Biological Cybernetics.

[32] Slotine JJE, Li W (1991). Applied nonlinear control..

[33] Utkin V (1992). Sliding modes in control and optimization.

[34] Loram ID, Maganaris CN, Lakie M (2005). Active, non-spring-like muscle movements in human postural sway: how might paradoxical changes in muscle length be produced?. Journal of Physiology.

[35] Stépán G, Kollár L (2000). Balancing with reflex delay.. Mathematical and Computer Modelling.

[36] Kloeden PE, Platen E (1992). Numerical solution of stochastic differential equations.

