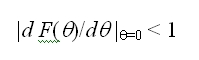# Correction: A Model of Postural Control in Quiet Standing: Robust Compensation of Delay-Induced Instability Using Intermittent Activation of Feedback Control

**DOI:** 10.1371/annotation/96e08e7f-22f0-445d-8fb3-fe7b071d0a3a

**Published:** 2009-07-31

**Authors:** Yoshiyuki Asai, Yuichi Tasaka, Kunihiko Nomura, Taishin Nomura, Maura Casadio, Pietro Morasso

In the Methods section, equation 11 is incorrect. Please view the correct equation here: